# Role of Dissimilative Pathway of *Komagataella phaffii* (*Pichia pastoris*): Formaldehyde Toxicity and Energy Metabolism

**DOI:** 10.3390/microorganisms10071466

**Published:** 2022-07-20

**Authors:** Julio Berrios, Chrispian W. Theron, Sébastien Steels, Belén Ponce, Edgar Velastegui, Cristina Bustos, Claudia Altamirano, Patrick Fickers

**Affiliations:** 1 School of Biochemical Engineering, Pontificia Universidad Católica de Valparaíso, Av. Brasil 2085, Valparaíso 2340000, Chile; belen.ponce@pucv.cl (B.P.); edgarvelasteguigonzalez@gmail.com (E.V.); cristinabustos_vero@hotmail.es (C.B.); claudia.altamirano@pucv.cl (C.A.); 2 GeneMill, Institute of Systems, Molecular and Integrative Biology, Biosciences Building, University of Liverpool, Crown Street, Liverpool L69 7BE, UK; c.theron@liverpool.ac.uk; 3 Microbial Processes and Interactions, TERRA Teaching and Research Centre, Gembloux Agro-Bio Tech, University of Liège, Av. de la Faculté 2B, 5030 Gembloux, Belgium; s.steels@uliege.be (S.S.); pfickers@uliege.be (P.F.)

**Keywords:** *Komagataella phaffii*, *Pichia pastoris*, methanol, formaldehyde dehydrogenase, dissimilative pathway

## Abstract

*Komagataella phaffii* (aka *Pichia pastoris*) is a yeast able to grow in methanol as the sole carbon and energy source. This substrate is converted into formaldehyde, a toxic intermediary that can either be assimilated to biomass or dissimilated to CO_2_ through the enzymes formaldehyde dehydrogenase (FLD) and formate dehydrogenase, also producing energy in the form of NADH. The dissimilative pathway has been described as an energy producing and a detoxifying route, but conclusive evidence has not been provided for this. In order to elucidate this theory, we generated mutants lacking the FLD activity (Δ*fld1*) and used flux analysis to evaluate the metabolic impact of this disrupted pathway. Unexpectedly, we found that the specific growth rate of the Δ*fld1* strain was only slightly lower (92%) than the control. In contrast, the sensitivity to formaldehyde pulses (up to 8mM) was significantly higher in the Δ*fld1* mutant strain and was associated with a higher maintenance energy. In addition, the intracellular flux estimation revealed a high metabolic flexibility of *K. phaffii* in response to the disrupted pathway. Our results suggest that the role of the dissimilative pathway is mainly to protect the cells from the harmful effect of formaldehyde, as they were able to compensate for the energy provided from this pathway when disrupted.

## 1. Introduction

**Figure 1:**
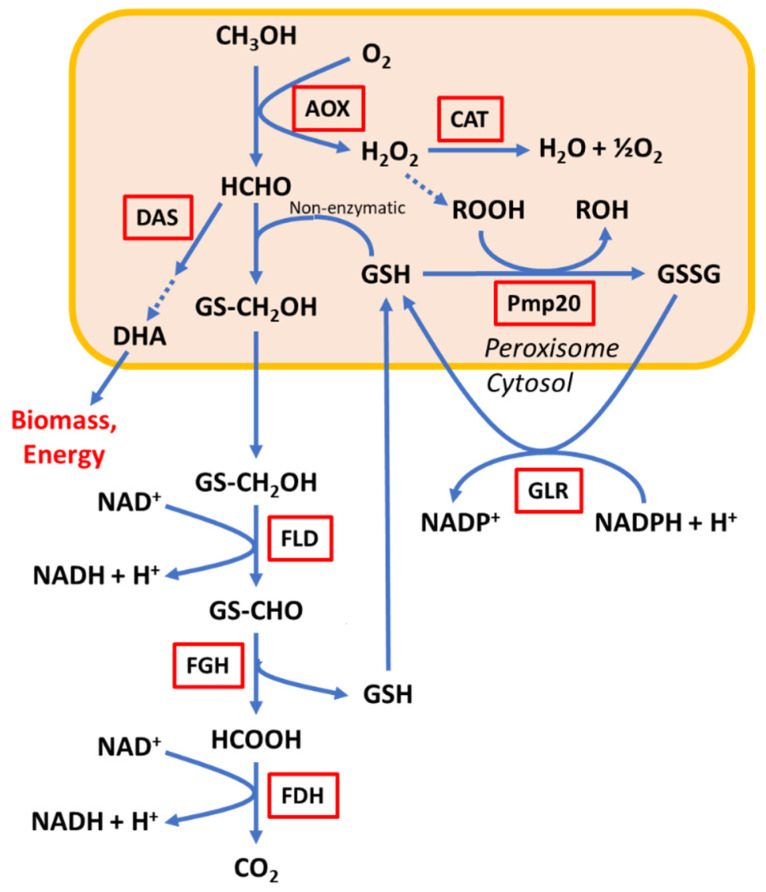
Main steps of the methanol utilisation (Mut) pathway in *K. phaffii*. Relevant enzymes involved are shown in red boxes. (Adapted from [10]). AOX: alcohol oxidase; CAT: catalase; DAS: dihydroxyacetone synthase; FLD: formaldehyde dehydrogenase; FGH: S-formylglutathione hydrolase; FDH; formate dehydrogenase; GS(H): glutathione; Pmp20: peroxisomal glutathione peroxidase; GLR: glutathione reductase.

*Komagataella phaffii* (formerly known as *Pichia pastoris*) is a methylotrophic yeast extensively used for recombinant protein production (See [1,2] for recent reviews). In this microorganism, methanol induces the peroxisome biosynthesis and expression of genes encoding enzymes required for its metabolism [3]. This alcohol is converted into formaldehyde from which two main branches are derived (Figure 1): The assimilative pathway that produces dihydroxyacetone (DHA) and connects to the glycolytic pathway for biomass synthesis and energy production; and the dissimilative pathway, where formaldehyde is oxidised ultimately to CO_2_, producing NADH during the process. Formaldehyde is a toxic intermediate whose accumulation may produce a detrimental effect on metabolism and cell growth by damaging DNA and RNA structures, among other effects [4]. This toxicity can be especially critical during cultures operated as fed-batch [5] or continuous cultures [6], where transient methanol concentration may suddenly increase as a result of the feeding flow, producing an imbalance between the formaldehyde production by the alcohol oxidase (AOX) and its consumption via either or both dihydroxyacetone synthase (DAS) or formaldehyde dehydrogenase (FLD). In this regard, Wakayama et al. [7] showed that activity of AOX in *P. methanolica* increased several hours after FLD and FDH when induced with methanol, revealing a protective mechanism to prevent formaldehyde accumulation in this methylotrophic yeast.

The role of the dissimilative pathway has been associated with both energy production and formaldehyde detoxification [8]. A previous report has shown that Δ*fld1* mutants of other methylotrophic yeast *Candida boidinii* [9] is unable to grow in methanol as a sole carbon source, highlighting the importance of the dissimilative pathway in this yeast. However, the existence of other pathways for energy production such as tricarboxylic acid cycle (TCAc) and oxidative phosphorylation suggests that these observations could have been caused by formaldehyde toxicity rather than lack of energy production. In fact, it is not clear that the energy obtained from the dissimilative pathway in *K. phaffii* is indeed essential for supporting cell viability and growth. To gain a deeper insight, we have studied the role of this pathway in methanol metabolism using the Δ*fld1* mutant of *K. phaffii*, estimating its energy contribution by means of a metabolic flux balance analysis and evaluating its connection to the toxic effect of formaldehyde.

## 2. Materials and Methods

### 2.1. Strains

The strains used in this study were *K. phaffii* RIY232 and a Δ*fld1* mutant RIY428 (Table 1). An FLD disruption cassette was constructed as previously described [11]. The P and T fragments (0.6 kb and 0.9 kb, respectively) corresponding of the 5′and 3′ end of the FLD1 gene were PCR amplified using primer pairs FLD1_PF/FLD1_PR and FLD1_TF/FLD1_TR, respectively, and the genomic DNA from the *K. phaffii* RIY232 strain as a template. Primers FLD1_PR and FLD1_TF have been designed to introduce an I*-Sce*I restriction site at 3′ and 5′ of the P and T fragments. After purification, P and T fragments were pooled and used as a template for PCR amplification using primer FLD_PF and FLD1_TR. The resulting fragment was cloned in pJet2.1 (ThermoScientific) and sequenced for verification purposes. The resulting vector RIP293 was used to introduce at I*-Sce*I restriction site 1.3 kb Zeocin resistance gene (Z*eoR*), rescued from vector RIE280. The resulting vector RIP293_ZeoR was digested by *Bgl*II and the 2.7 kb purified P-ZeoR-T was used to transform the *K. phaffii* RIY232 strain as described by Lin-Cereghino et al. [12]. The correct FLD disruption was verified by PCR using primer FLD1_Verif and FLD1_TR. Primer FLD1_Verif hybridise 250 bp upstream (at 5′ end of *FLD1*) as compared to FLD1_PF. The resulting strain was named RIY428 (Δ*fld1* strain). All molecular biology techniques were as described in Theron et al. [13].

### 2.2. Cell Cultures and Formaldehyde Pulses

Unless stated otherwise, cultures were performed in triplicate using 500 mL shake flasks (30 °C, 200 rpm) with 100 mL of the defined medium Yeast Nitrogen Base (YNB) without amino acids, supplemented with ammonium sulphate (5 g/L) and either methanol or glycerol (3 g/L) as the only carbon source. Flasks were inoculated with each strain to an initial biomass concentration of 0.3 g/L using washed cells previously grown in 3 g/L of the same carbon source (either methanol or glycerol) employed in the respective experiment. Samples (1 mL) were taken regularly to follow biomass production and methanol consumption. Formaldehyde pulses were made 4 h after inoculation and performed by adding the required volume for final concentration of 2, 5 or 8 mM.

### 2.3. Parameter Calculation

Specific growth rate (*µ*) was estimated from the positive slope in the linear region (R^2^ > 0.98) of the semi log plot of biomass concentration (*X*) vs. time [14]. Correspondingly, negative slope was used to estimate the cell death constant (*k_d_*).

The yield of substrate-into-biomass (*Y_XS_*) was estimated from the slope in the linear zone (R^2^ > 0.98) of the methanol concentration (*S*) vs. *X*, according to Equation (1):

(1) $$Y_{\mathrm{XS}}=\frac{\Delta X}{-\Delta S}$$

The specific consumption rate of substrate (*q_S_*) was estimated from the Equation (2) in the range where both the specific growth rate of biomass (*µ*) and *Y_XS_* were constant (balanced growth):

(2) $$q_{S}=\frac{\mu }{Y_{XS}}$$

The formaldehyde effect on *µ* was quantified by estimating the inhibition constant (*K_i_*). For this, we have used the Monod equation and assumed a growth substrate inhibition kinetics [15]. An additional inhibition expression for the inhibition effect of formaldehyde concentration (*Form*) was included:

(3) $$\mu =\mu_{max}\;\left(\frac{S}{K_{S}+S+\frac{S^{2}}{K_{i,S}}}\right)\left(\frac{K_{i}}{\left[Form\right]+K_{i}}\right)$$

where *K_S_* is the substrate saturation constant of the Monod equation, and *K_i,S_* is the inhibition constant of the substrate. Reference values of these constants can be found in Canales et al. [15]. In a previous report we found that no significant changes in *µ* were observed when methanol concentration ranged between 1.0 and 4.0 g/L in shake flask cultures [15]. Thus, *S* was experimentally set within the range where it was higher than the substrate constant (*S* >> *K_S_*), but without a relevant inhibition effect (*S* >> *S^2^*/*K_i,S_*). This was experimentally verified in the period where a constant *µ* was observed; meanwhile, methanol concentration varied within the range of 3.0 to 2.0 g/L due to cell consumption (i.e., when the specific growth rate was independent from methanol concentration). Under these experimental conditions, Equation (3) can be simplified to:

(4) $$\mu =\mu_{max}\;\left(\frac{K_{i}}{\left[Form\right]+K_{i}}\right)$$

The minimisation of mean square error (MSE) between the predicted values and experimental data was used for estimating *K_i_* and *μ_max_* from Equation (4), according to the following equation:

(5) $$\mathrm{Min}:\;\mathrm{MSE}=\frac{1}{n}\overset{n}{\underset{j=1}{\sum }}{\left({\hat{\mu }}_{j}-\mu_{j}\right)}^{2}$$

where $\hat{\mu }$ represents the model estimation of experimental *µ* values. Optimisation was performed using the SOLVER tool in Microsoft Excel.

The relationship between *q_S_* and *µ* was evaluated by estimating their linear correlation according to the following equation:

(6) $$q_{S}={m^{\prime }}_{S}+\frac{\mu }{{Y^{\prime }}_{XS}}$$

Equation (6) is analogous to that described for estimating the maintenance energy coefficient *m_S_* [16], but here, both *Y′_XS_* and *m′_S_* are apparent parameters obtained under energy-sufficient growth that consider a growth-rate dependent maintenance energy coefficient as described by Pirt [17].

### 2.4. Metabolic Flux Analysis

Metabolic fluxes for each strain were calculated by means of a stoichiometric model for *K. phaffii*, as reported previously [18]. The original model was reduced to 40 reactions that included main pathways for methanol metabolism, glycolysis and gluconeogenesis, TCAc, pentose phosphate pathway (PPP) and biomass biosynthesis (See Supplementary File S1). The metabolic information for the reaction network was obtained from previous metabolic flux models [19,20,21] and lumped when possible in order to reduce the size of the stoichiometric matrix. The reaction for biomass formation considered the contribution of the macromolecular composition (proteins, lipids, polysaccharides, DNA and RNA) and balanced to obtain the empiric biomass formula CH_1.89_N_0.137_O_0.785_, as it was obtained from the *K. phaffii* elemental composition [22,23]. The model was solved using the software MetaFluxNet [24,25,26], minimising the flux of ATP for maintenance (Reaction 39, Supplementary File S1) under the assumption that, for a given specific growth rate and unlimited substrate cell growth condition, cells synthesise new cells at the lowest energy consumption [27]. The ATP flux for maintenance has been successfully used in several stoichiometric flux models as a way to represent any metabolic process that is occurring in the cell that requires energy (e.g., intracellular pH homeostasis), in addition to the biomass formation [18,20,28]. All fluxes were expressed in mmol/gDCW h.

### 2.5. Analytical Methods

The biomass was determined by optical density to be 600 nm and it was converted to dry cell weight (DCW) using a calibration curve as previously described [15]. Methanol concentration was measured by gas chromatography (GC-FID) in a Clarus 600 chromatograph (PerkinElmer) using a Supelco Equity-1 capillary column with 5 mL/min of N_2_ as mobile phase, using 200, 80, and 200 °C as the temperature for the injector, column, and detector, respectively.

### 2.6. Satatistical Analysis

Unless otherwise stated, kinetic parameters and yields were calculated from measurements obtained in three independent experiments. Where suitable, values are expressed as mean ± standard error of the mean (SEM) or as a percentage of the mean. An unpaired *t*-test was used to compare the means using the software GraphPad InStat version 3.05 (Graphpad Software Inc., San Diego, CA, USA). The threshold for statistical significance was *p* < 0.05.

## 3. Results and Discussion

### 3.1. Effect of FLD1 Gene Deletion on Cell Growth

**Figure 2:**
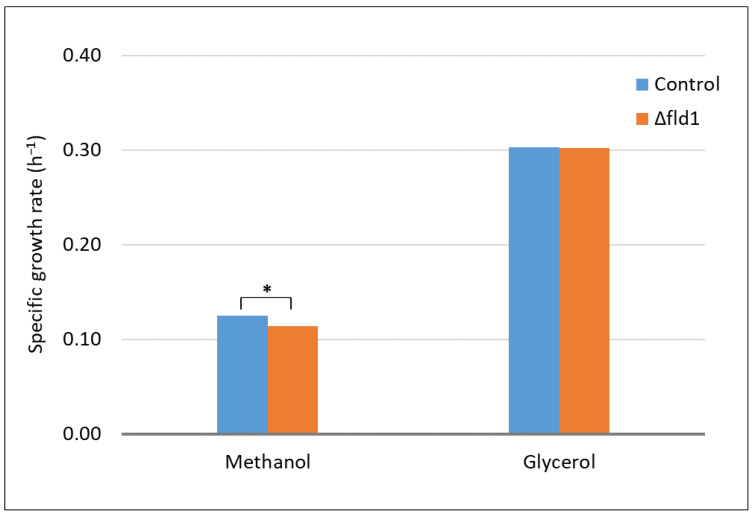
Specific growth rate (*µ*) of control and Δ*fld1* strains grown on a defined medium using either methanol or glycerol 3 g/L as the only carbon source. * Significant difference evaluated by an unpaired *t*-test * *p* < 0.01. Cell growth profiles are shown in Supplementary Figures S2 and S3.

In order to evaluate the role of the dissimilatory pathway of *K. phaffii* in methanol metabolism and formaldehyde detoxification, the *FLD1* gene encoding formaldehyde dehydrogenase was disrupted in strain GS115. The specific growth rate (*µ*) was estimated for Δ*fld1* and control strains in either methanol or glycerol as the only carbon source (Figure 2). Remarkably, unlike the Δ*fld1* mutant of *C. boidinni* [9], the Δ*fld1* strain of *K. phaffii* was able to grow in methanol (*µ_max_* = 0.114 ± 0.003 h^−1^) at a similar (but significantly lower) specific growth rate of the non-disrupted control strain (*µ_max_* = 0.125 ± 0.001 h^−1^). In contrast, using glycerol as a carbon source lead to the same *µ_max_* (0.3 h^−1^) with Δ*fld1* and control strains (Figure 2), in agreement with previous reports obtained in this carbon source [14]. As expected, this result indicates that the disrupted gene only affected the metabolism of methanol. This ability of *FLD*-lacking cells to grow on cultures with methanol as the only carbon source has also been reported in *Hansenula polymorpha* [29]. In those experiments, cultures were performed using 4 g/L methanol. In contrast, the absence of cell growth reported in the Δ*fld1* mutant of *C. boidinii* were conducted with 11.9 g/L methanol [9]. In order to explore if high methanol concentrations could prevent cell growth in cultures of the Δ*fld1* strain of *K. phaffii*, we performed cultures of *K. phaffii* using 11.9 g/L methanol, confirming that both the control and Δ*fld1* strains were able to grow at higher methanol concentrations after 36 h (Supplementary Figure S1). This finding indicates that the absence of growth observed in Δ*fld1* mutants of *C. boidinii* cannot be explained by high methanol concentration. Further research is required to provide the evidence needed.

Although the disruption of the *FLD1* gene has been previously reported in *K. phaffii*, those cultures were performed using complex media [10], making it impossible to elucidate the cell growth of these Δ*fld1* mutants with methanol as the only carbon source. Tyurin & Kozlov [30] reported an experimental procedure using minimal agar medium containing methanol as the only carbon source. However, the results shown were also performed using a complex medium. In addition, Shen et al. [31] had reported the *K. phaffii* strain defective in the *FLD1* gene that was not able to grow in 4 g/L methanol as the only carbon source. The strain obtained by random mutagenesis was also reported to have other relevant enzymes activities for methanol metabolism severely reduced (including *AOX*, *CAT*, *FDH*, *and DAS* in Figure 1). The lack of selectivity on random genetic modifications could explain the absence of growth observed in that report, in contrast to what we have observed here.

### 3.2. Evaluation of Formaldehyde Toxicity 

**Figure 3:**
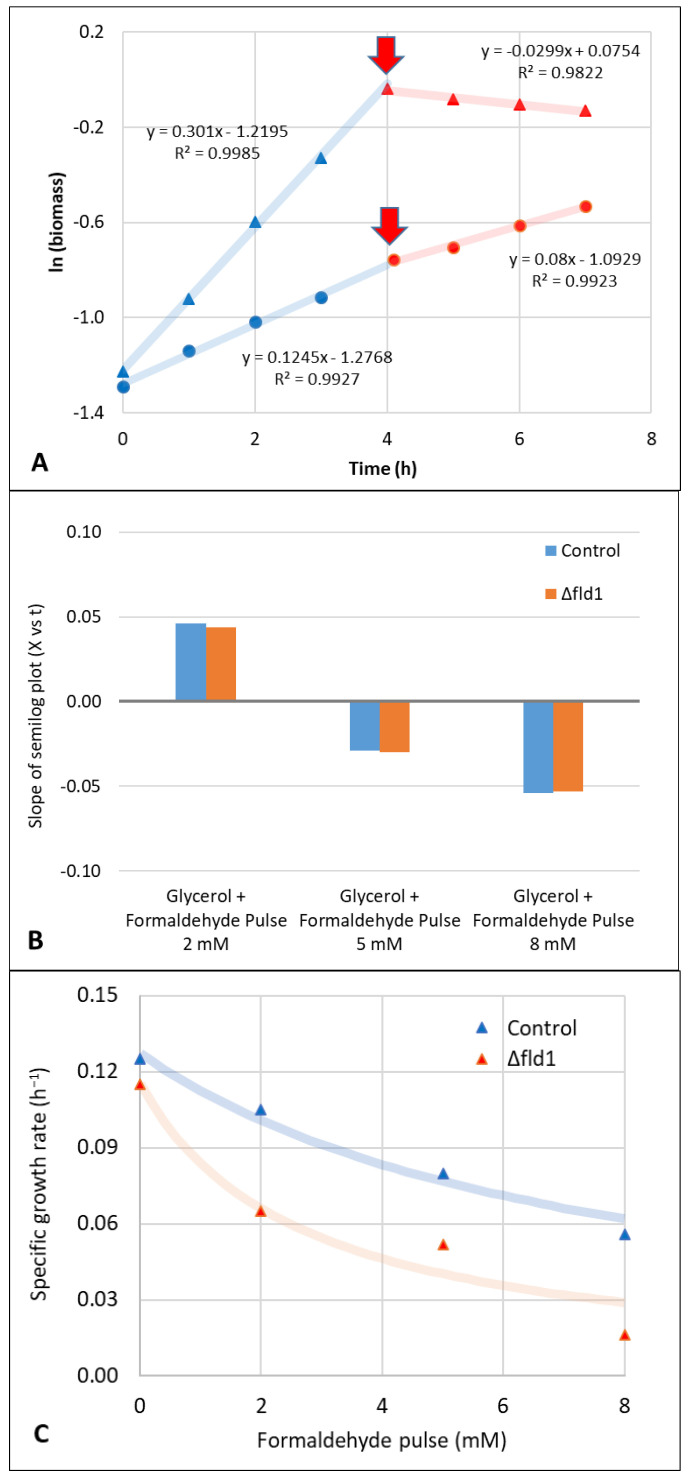
(**A**) Example of biomass growth kinetics (shown as ln*X*) of control strain with glycerol (triangles) or methanol (circles) as the carbon source. The growth behaviour before (blue symbols) and after (red symbols) a formaldehyde pulse (5 mM) performed after 4 h of inoculation (red arrow) is shown. The experimental error in biomass measurements was lower than 3% (not shown). (**B**) The effect of formaldehyde pulses (2, 5 and 8 mM) on the cell growth kinetics (slope of semi log plot) of control (blue) and Δ*fld1* (orange) strains using glycerol as the only carbon source. (**C**) The compared effect of formaldehyde pulses (2, 5 and 8 mM) on control (blue triangles) and Δ*fld1* (red triangles) strains using methanol as the only carbon source. Model adjustment Equation (5) is also shown with continuous lines for each strain. SEM < 5% (not shown).

The toxic effect of formaldehyde on cell growth using either methanol or glycerol as carbon source was evidenced by the change in the specific growth rate caused by pulses of this compound after 4 h of inoculation (Figure 3A). A severe effect of these pulses was observed in glycerol cultures (Figure 3B), reducing *µ* by 85% after a formaldehyde pulse of 2 mM (Figure 3B). Furthermore, pulses of higher concentrations (5 or 8 mM) caused a decrease in biomass concentration. The values of *k_d_* calculated from the negative slope of the ln *X* vs. time plot were 0.030 and 0.054 h^−1^, respectively (Figure 3B). Interestingly, no significant differences were observed between Δ*fld1* and the control strains.

A lower effect of formaldehyde pulses on *µ* was verified in methanol cultures when compared to glycerol cultures, but significant differences were observed between control and Δ*fld1* strains, with a lower tolerance of the later evidenced by a more pronounced reduction of *µ* (Figure 3C). The quantitative effect is properly described by the formaldehyde inhibition constant (*K_i_*) that with the Δ*fld1* strain displayed a value that was approximately half the control strain (7.57 mM). This indicates that the inhibition effect in the disrupted strain occurs at a lower formaldehyde concentration than the control. Yano et al. [10] reported a Δ*fld1* strain grown in glucose as a carbon source to be hypersensitive to formaldehyde 2mM. Other formaldehyde consuming enzymes such as DAS are normally repressed in the presence of glucose, probably explaining the reason for the toxic effect observed even after 48 h of culture [8]. The synthesis of methyl formate in some methylotrophic yeasts such as *C. boidinii* [32] and *P. methanolica* [33] has been proposed as an alternative for formaldehyde detoxification. This reaction can be catalysed by both methyl formate synthase and alcohol dehydrogenases. However, to our knowledge, methyl formate synthesis has not been reported in *K. phaffii*. Moreover, recent genome-scale metabolic models of *K. phaffii* have not included this intermediary in their reaction networks containing more than a thousand metabolites [34,35], suggesting that methyl formate is not involved in formaldehyde detoxification in *K. phaffii*. Further research is required to verify this hypothesis.

Formaldehyde toxicity has also been evaluated in other methylotrophic yeasts such as *P. methanolica*, where concentrations up to 5 mM had a major impact on cell growth [7]. The dynamic balance between the expression of *AOX* and *FLD* genes proved to be a key factor in cell prevention of formaldehyde toxicity. On the other hand, glycerol cultures showed a higher sensitivity to formaldehyde than methanol cultures (Figure 3). This behaviour has been also observed in *P. methanolica* and has been explained by the presence of the DAS enzyme that is induced by methanol (but not by glycerol) and metabolised the formaldehyde, reducing its toxic effect [7]. Formaldehyde pulses were performed in the extracellular medium, while the main metabolic routes involved in its consumption are either in the cytoplasm (dissimilative pathway) or the peroxisome (assimilative pathway). Being a small polar non-charged molecule that can freely diffuse across cell membranes [36], the effect of formaldehyde pulses can reach the whole cell (including its organelles). Thus, fast diffusion explains the growth rate reduction because of the extracellular pulse (Figure 3).

The specific consumption rate of methanol (*q_S_*) in batch cultures was estimated to be within the range where balanced growth was verified, i.e., when both *µ* and *Y_XS_* were constant. This was observed for at least 4 h before and after the formaldehyde pulse (Figure 3A). For simplicity, we only show the effect at higher formaldehyde concentrations (5 and 8 mM) on both the control and Δ*fld1* strains. The values of *Y_XS_* and *q_S_* in the reference condition, i.e., without the formaldehyde pulse (Table 2) are similar to those previously reported using methanol as the sole and non-limiting carbon source [37,38]. On the other hand, *q_S_* was the highest in the absence of formaldehyde for both strains, and they were considerably reduced by the effect of formaldehyde pulses. In addition, while this compound slightly affected *Y_XS_* of the control, a marked effect was observed with Δ*fld1* strain. Since *Y_XS_* represents the fraction of consumed carbon source that is converted into biomass, the lower the value the higher the methanol fraction that is diverted to fates other than biomass. It has been shown that microorganisms consume energy to maintain their cellular integrity and homeostasis, including conditions of stress [16]. Based on this, our results suggest that, as a response to formaldehyde pulse, methanol is being consumed to produce maintenance energy instead of biomass.

### 3.3. Maintenance Energy Estimation

**Figure 4:**
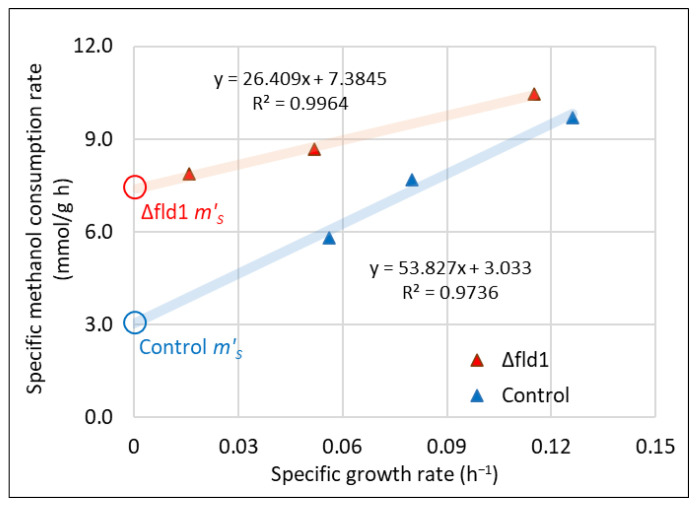
Correlation between the specific consumption rate of methanol (*q_S_*) and the specific growth rate (*µ*) for control (blue) and Δ*fld1* (red) stains. The apparent maintenance coefficient *m′_S_* was estimated from Equation (6). Control: blue open circle; Δ*fld1* strain: red open circle.

For a better insight into the methanol utilisation after the formaldehyde pulses, we have explored the relationship between *q_S_* and *µ* (Figure 4). Remarkably, we have found a strong linear correlation (R^2^ > 0.97) between these two variables, with different slopes and y-axis intercepts for each strain. This trend matches the previously described behaviour associated with the growth-dependent maintenance energy described by Pirt [17] which was observed under energy-sufficient growth conditions, i.e., not limited by energy sources in the cell culture. A detailed description of the parameter definition under those conditions is described in [17]. For simplicity, we have condensed these parameters into the apparent yield (*Y′_XS_*) and apparent maintenance coefficient (*m′_S_*) as shown in Equation (6). Here, *μ* is a function of formaldehyde concentration (as is also shown in Equation (4)) instead of nutrient concentration. The value of *m′_S_* for Δ*fld1* strain was 2.5-fold higher than control strain (7.4 compared to 3.0 mmol/ g_DCW_·h), revealing a higher demand of maintenance energy in the Δ*fld1* strain in the presence of formaldehyde. This finding suggests that the lack of dissimilative pathway changes the ability of cells to manage the toxic effects of formaldehyde in terms of maintenance energy. This was explored more deeply by means of a metabolic flux estimation.

### 3.4. Metabolic Flux Analysis

**Figure 5:**
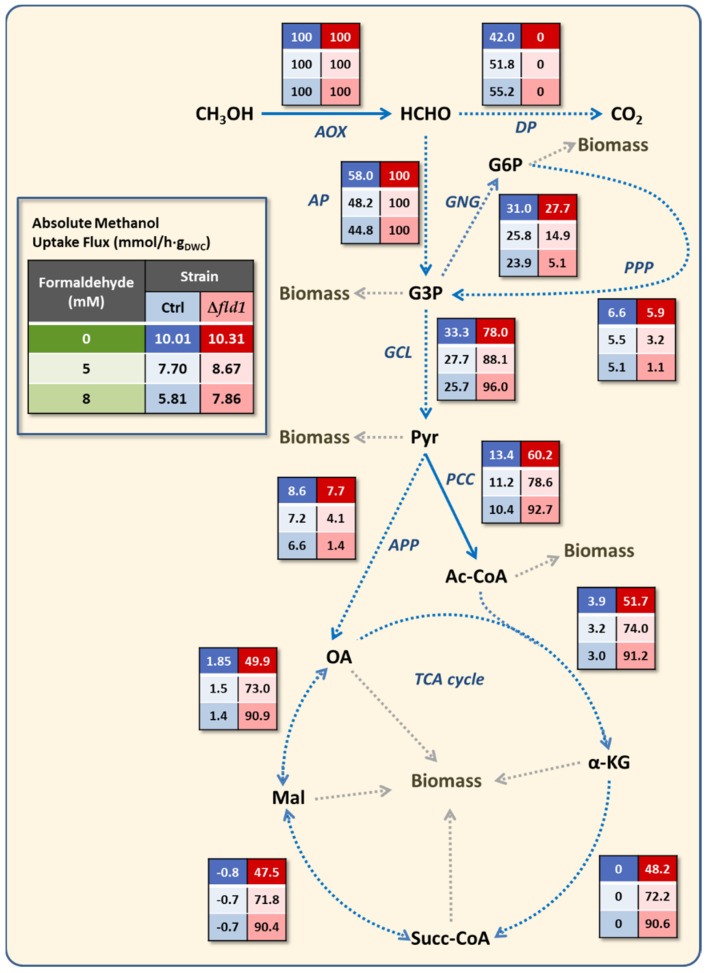
Simplified map of the metabolic flux distribution of methanol uptake in *K. phaffii* (blue columns: control strain; red columns: Δ*fld1* strain). The list of the metabolic reactions is provided in Supplementary File S1. Results are shown as percentage of the methanol uptake flux with no pulse (0) and after 5 or 8 mM formaldehyde pulse (rows). AOX: alcohol oxidase; DP: dissimilative pathway; AP: assimilative pathway; GNG: gluconeogenesis; GLC: glycolysis (from G3P onwards); PDH: pyruvate dehydrogenase; APP: anaplerotic pathways; G3P: glycerdaldyde-3-phosphate; G6P: glucose- 6-phosphate; Pyr: pyruvate; Ac-CoA: actyl coenzime A; α-KG: α -ketoglutarate; Succ-CoA: succinyl conezyme A; Mal: malate; OA: oxaloacetate.

A deeper study on the metabolism *K. phaffii* performed through a metabolic flux analysis, using a stoichiometric model previously described [18]. Carbon flux distribution was estimated, and the effect of disrupting the *FLD1* gene and formaldehyde pulses were contrasted. Fluxes through both the dissimilative and assimilative pathways in the control strain decreased as a consequence of formaldehyde pulses compared to the reference condition (Table 3). This reduction is related to the decrease of methanol flux described above. The flux distribution through these pathways can also be seen as a percentage of methanol uptake flux in Figure 5. The percentage of carbon source directed to the assimilative pathway ranged from 60% without formaldehyde to 48% after 8mM pulse, demonstrating that both assimilative and dissimilative pathways remain active when exposed to this toxic compound.

**Figure 6:**
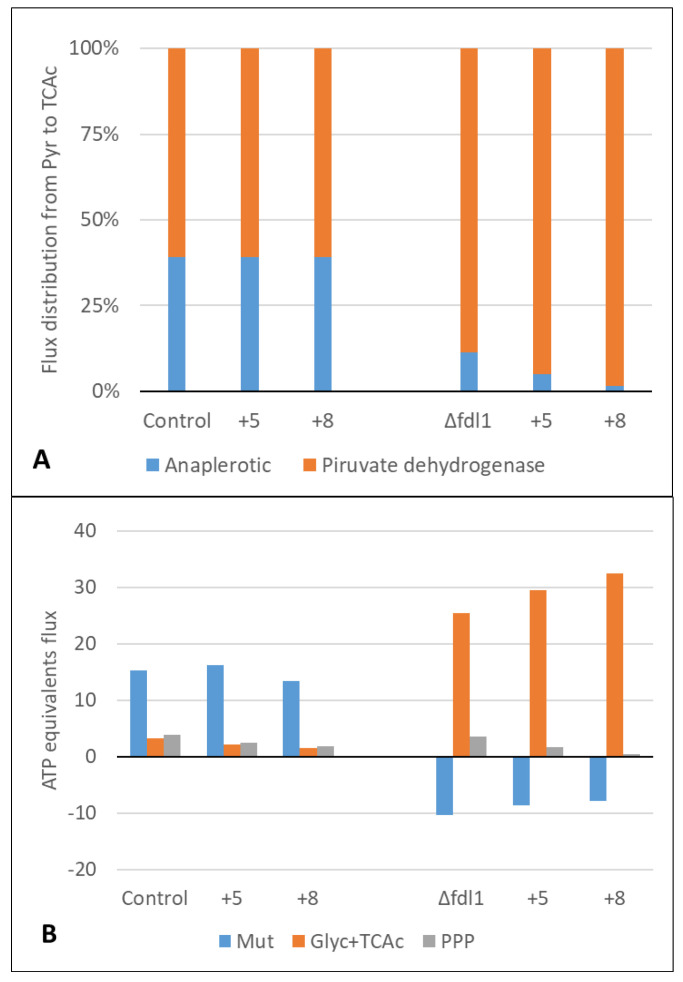
(**A**) Carbon flux distribution from pyruvate (Pyr) to the TCAc through the anaplerotic pathway or pyruvate dehydrogenase (**B**) Net flux of ATP equivalents flux (in mmol/g_DCW_·h) produced (positive values) or consumed (negative values) in control and Δfld1 strains. Mut: methanol utilisation pathway; Glyc+TCAc: glycolysis plus TCAc; PPP: pentose phosphate pathway.

The disruption of this node in the Δ*fld1* strain not only redirected the total flux of carbon through the assimilative pathway but also caused a flux redistribution toward other relevant nodes such as pathways derived from pyruvate (Figure 5). However, the flux fraction from this intermediate to TCAc was not affected by formaldehyde in the control strain but was remarkably increased to the detriment of the anaplerotic pathway in the Δ*fld1* strain (Figure 6A). The later has a relevant role in replenishing the TCAc intermediaries when they are used for biosynthesis. In contrast, the TCAc is directly related to energy synthesis through the generation of GTP, NADH and FADH_2_. This change in flux distribution seems to agree with the abovementioned observation of maintenance energy requirements in the Δ*fld1* strain, as higher flux to the TCAc could be related to higher energy demands from the cell.

In order to confirm if the energy demand is increased by the lack of a dissimilative pathway, we have calculated the flux of ATP for maintenance by including the consumption and/or production of ATP (or GTP), NAD (P) H and FADH_2_ in the metabolic reactions of the stoichiometric model. The estimation of the energy associated with maintenance has been used in several modelling approaches in yeast, including stoichiometric models to represent the metabolic processes that are occurring in the cell for viability and maintenance of homeostasis [18,20,28]. Results show that the ATP flux for maintenance was not affected by 5 mM formaldehyde pulses in the control strain, while a 20% reduction was observed after the 8 mM formaldehyde pulse (Table 3). In contrast, this flux was higher in the Δ*fld1* strain compared to the control, and it increased even more after the formaldehyde pulse to double the value of the control strain after the 8 mM formaldehyde pulse. This means that there is a higher specific energy flux used for maintenance as a result of the disruption of the dissimilative pathway.

Based on these results, the estimation of net energy contribution of the Mut pathway, glycolysis and TCAc, and PPP was made (Figure 6B). Results showed that the Mut pathway is the main source of ATP equivalents in the control strain, even when exposed to formaldehyde pulses. This is energy is mainly provided as reducing power by the dissimilative pathway that is fully active in this strain. In contrast, the Mut pathway in Δ*fld1* does not produce but rather consumes net energy, since no NADH is produced, while at the same time ATP is required for phosphorylation of dihydroxyacetone by the enzyme dihydroxyacetone kinase in the assimilative pathway [39]. In addition, it was observed that the main source of energy in this mutant strain is associated with the glycolytic pathway (from dihydroxyacetone phosphate onwards) and TCAc (Figure 6B). In this regard, a proteomic analysis of *K. phaffii* GS115 showed that the increased amount of enzymes of the Mut pathway caused by methanol induction occurred together with a decrease of fumarase and aconitase in the TCAc [5]. Additionally, Russmayer et al. [8] described an inverse relationship between the methanol dissimilation and the TCAc flux, comparing cultures growing with methanol/glycerol and glucose. A recent report with the methylotrophic yeast *Ogataea methanolica* indicates that TCAc was up or downregulated as a response to intracellular level of acetyl-CoA associated with the methanol concentration [40], suggesting an metabolic adaptation capacity of this yeast to maintain its energy status. In agreement, our evidence in *K. phaffii* indicates that cells exhibit a metabolic flexibility to generate reducing power. In the present case, cells lacking the dissimilative pathway were able to fulfil the energy requirements through pathway flux rearrangement at glycolytic and TCAc levels. Besides this, formaldehyde pulses had a major impact on flux distribution in the Δ*fld1* strain, which is mainly caused by its toxicity.

## 4. Conclusions

In this work, we have demonstrated that the *FLD1* gene is not essential for cell viability and the growth of *K. phaffii* when methanol is the only carbon and energy source, in contrast with a previous report on Δ*fld1* strains obtained by random mutagenesis. Our results revealed a high metabolic flexibility of *K. phaffii* in response to the disrupted dissimilative pathway, as the Δ*fld1* strain was able to produce energy to fulfil their requirements and compensate for the energy that is normally produced in this pathway. Despite this metabolic flexibility, the toxic effect of formaldehyde was significantly increased in the Δ*fld1* strain, supporting the idea that the dissimilative pathway is a formaldehyde detoxifying route.

## Figures and Tables

**Table 1:** Plasmids primer and strains used in this study.

Strains	Genotype-Plasmid	Source/Reference
*E. coli* DH5α	Δ(*lac*ZYA-*arg*F)U169 *rec*A1 *end*A1 *hsd*R17(rK–, mK+) *pho*A *sup*E44 λ– *thi*-1 *gyr*A96 *rel*A1 F– φ80lacZΔM15	Promega
RIE280	DH5α, vector RIP280 (I-*Sce*I -ZeoR- I-*Sce*I)	Lab stock
RIE293	DH5α, vector RIP293 (P_FLD1_- I*-Sce*I -T_FLD_ in pJet2.1)	This work
RIE293_ZeoR	DH5α, vector RIp293_ZeoR (P_FLD1_-ZeoR-T_FLD_)	This work
RIY232	*Komagatella phaffii GS115, HIS4* (protothroph)	Lab stock
RIY428	RIY232, *FLD1::ZeoR*	This work
Primer	Primer sequences 5′-3′	Modification
FLD1_PF	TACACAACGGATGTCGCACT	
FLD1_PR	CAGGAAACAGCTATGACCCGAACACAACAGGGAAACT	I-*Sce*I
FLD1_TF	GTAAAACGACGGCCAGTTGGCAGAGTCTGGAGAGGAT	I-*Sce*I
FLD1_TR	GAGATCCCAGGCATTCAGAG	
FLD1_verif	GGCACGGTGCTAATGGTAGT	

**Table 2:** Effect *FLD1* gene deletion on yield of methanol into biomass and the specific consumption rate of methanol *.

	Formaldehyde Pulse (mM)					
	0		5		8	
Strain	*Y_XS_*	*q_s_*	*Y_XS_*	*q_s_*	*Y_XS_*	*q_s_*
Control	0.38 **	10.01 **	0.32	7.70	0.31	5.81
Δ*fld1*	0.35	10.31	0.19	8.67	0.06	7.86

* *Y_XS_* in g_DCW_/g; *q_s_* in mmol/g_DCW_·h ** Reference condition

**Table 3:** The effect of *FLD1* deletion and formaldehyde pulses on flux distribution of between assimilative and dissimilative pathways and ATP flux for maintenance (mmol/g_DCW_ h).

	Control			Δfld1		
Formaldehyde Pulse (mM)	0 *	5	8	0	5	8
Dissimilative pathway	4.21	3.98	3.21	-	-	-
Assimilative pathway	5.80	3.72	2.60	10.31	8.67	7.86
ATP flux for maintenance	18.5	18.7	14.9	21.0	28.1	32.9

* Reference condition

## Supplementary Materials

The following supporting information can be downloaded at: https://www.mdpi.com/article/10.3390/microorganisms10071466/s1, File S1: List of Metabolic Reactions; File S2: Figure S1. Growth with high methanol concentration; Figure S2. Growth profile in glycerol; Figure S3. Growth profile in methanol.
