# Supplementary material for: Role of Dissimilative Pathway of Komagataella phaffii (Pichia pastoris): Formaldehyde Toxicity and Energy Metabolism
Source: Microorganisms. 2022 Jul 20;10(7):1466. doi: 10.3390/microorganisms10071466 (PMC9321669; doi:10.3390/microorganisms10071466)
Supplement: Supplementary file 1 [file microorganisms-10-01466-s001.zip › Suppl File S2.pdf]

Supplementary Information:

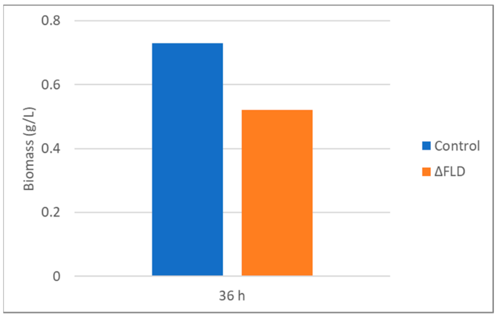

Figure S1. Biomass growth of control and *Δfld1* strains of *K. phaffii* obtained after 36h culture in define media using 11.9 g/L methanol (1.5% v/v) as the only carbon source. A 0.3 g/L inoculum using cells previously grown in 3 g/L methanol was utilised. Two independent replicates for each strain were performed (SEM < 0.02 g/L)

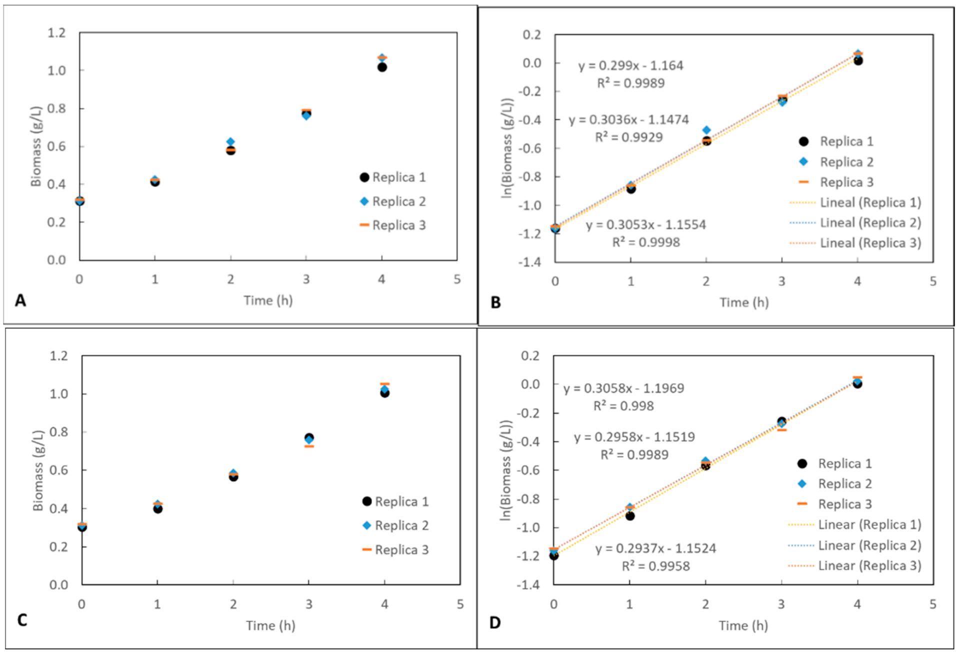

Figure S2. Biomass growth profiles obtained in define media using glycerol 3 g/L as the only carbon source (A) Growth profile for the control strain. (B) ln(biomass concentration) vs time for the control strain. Linear regression and equation for each replica is also included. (C) Growth profile for the *Δfld1* strain. (D) *ln*(biomass concentration) vs time for the *Δfld1* strain. Linear regression and equation for each run is also included. Specific growth rate (μ) values are shown in Figure 2 in the article.

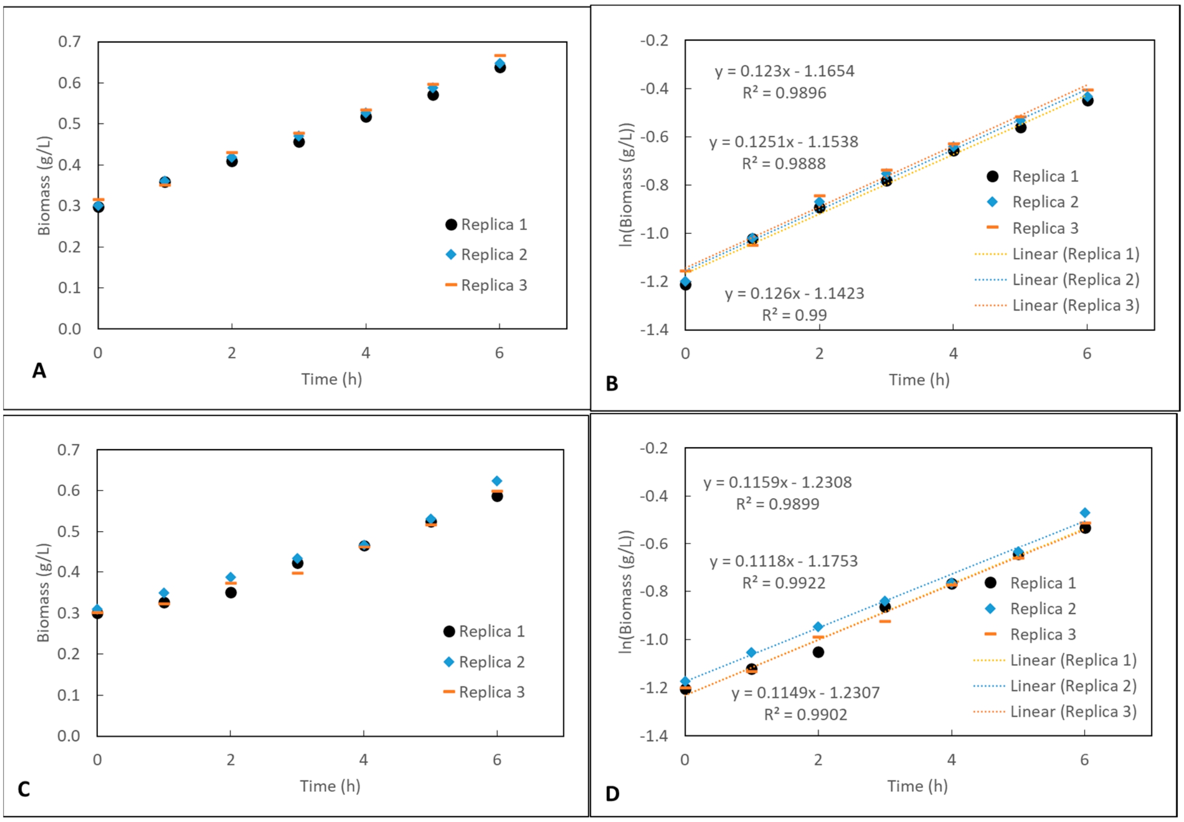

Figure S3. Biomass growth profiles obtained in define media using methanol 3 g/L as the only carbon source (A) Growth profile for the control strain. (B) ln(biomass concentration) vs time for the control strain. Linear regression and equation for each replica is also included. (C) Growth profile for the *Δfld1* strain. (D) *ln*(biomass concentration) vs time for the *Δfld1* strain. Linear regression and equation for each run is also included. Specific growth rate (μ) values are shown in Figure 2 in the article.
